# What, why and how do health systems learn from one another? Insights from eight low- and middle-income country case studies

**DOI:** 10.1186/s12961-018-0410-1

**Published:** 2019-01-21

**Authors:** Sophie Witter, Ian Anderson, Peter Annear, Abiodun Awosusi, Nitin N. Bhandari, Nouria Brikci, Blandine Binachon, Tata Chanturidze, Katherine Gilbert, Charity Jensen, Tomas Lievens, Barbara McPake, Snehashish Raichowdhury, Alex Jones

**Affiliations:** 1 ReBUILD and Institute for Global Health and Development, Queen Margaret University Edinburgh, Musselburgh, EH21 2UU United Kingdom; 2 Crawford School of Public Policy, Australian National University, Canberra, Australia; 3 Nossal Institute for Global Health, University of Melbourne, Melbourne, Australia; 4 Oxford Policy Management (OPM), Oxford, United Kingdom

**Keywords:** Policy transfer, Evidence use, Health systems, Learning, Low-income countries

## Abstract

**Background:**

All health systems struggle to meet health needs within constrained resources. This is especially true for low-income countries. It is critical that they can learn from wider contexts in order to improve their performance. This article examines policy transfer and evidence use linked to it in low- and middle-income settings. The objective was to inform international investments in improved learning across health systems.

**Methods:**

The article uses a comparative case study design, drawing on case studies conducted in Bangladesh, Burkina Faso, Cambodia, Ethiopia, Georgia, Nepal, Rwanda and Solomon Islands. One or two recent health system reforms were selected in each case and 148 key informants were interviewed in total, using a semi-structured tool focused on different stages of the policy cycle. Interviewees were selected for their engagement in the policy process and represented political, technical, development partner, non-governmental, academic and civil society constituencies. Data analysis used a framework approach, allowing for new themes to be developed inductively, focusing initially on each case and then on patterns across cases.

**Results:**

The selected policies demonstrated a range of influences of externally imposed, co-produced and home-grown solutions on the development of initial policy ideas. Eventual uptake of policy was strongly driven in most settings by local political economic considerations. Policy development post-adoption demonstrated some strong internal review, monitoring and sharing processes but there is a more contested view of the role of evaluation. In many cases, learning was facilitated by direct personal relationships with local development partner staff. While barriers and facilitators to evidence use included supply and demand factors, the most influential facilitators were incentives and capacity to use evidence.

**Conclusions:**

These findings emphasise the agency of local actors and the importance of developing national and sub-national institutions for gathering, filtering and sharing evidence. Developing demand for and capacity to use evidence appears more important than augmenting supply of evidence, although specific gaps in supply were identified. The findings also highlight the importance of the local political economy in setting parameters within which evidence is considered and the need for a conceptual framework for health system learning.

## Background

All health systems face challenges managing complex and changing health needs, with these challenges being the greatest in low-income countries due to the larger health needs, faster population growth [1] and least availability of financial resources for health [2]. At the same time, these governments are committed to progressing towards universal health coverage as part of the Sustainable Development Goals [3], within a context of more constrained development assistance [4, 5]. The use of evidence from other countries may result in health system reforms that are more efficient and effective [6–8]. This study seeks to understand policy transfer and evidence use around health systems in low- and middle-income settings in order to inform investments in improved learning between countries.

Globalisation and the activity of international organisations involved in the design, implementation and analysis of regional and domestic policies have facilitated dialogue and sharing of ideas and experiences across actors in different settings. The process of using the ideas, content and lessons from implementing policy from other countries, or what this study terms ‘learning across systems’, falls under the broader literature on policy transfer. Transfer is defined as the intentional process through which “*knowledge about policies, administrative arrangements, institutions etc. in one time and/or place is used in the development of policies, administrative arrangements and institutions in another time and/or place*” [9]. Unintentional emulation of policies, on the other hand, may be considered to be merely a ‘convergence’ of policy rather than a process in which one actor deliberately seeks and uses lessons from other actors [10].

A small but growing set of literature seeks to understand policy transfer processes in the health sector of low-income countries. Mechanisms of policy transfer that are identified include learning, coercion, socialisation and competition [11]. Financial assistance, identified as the most dominant form of coercion, has also led to changes in in-country policy, in many cases the adaptation of policy specifically to receive aid [12]. Significant attention in the literature has been placed on the role of international organisations, while questions around individual country-to-country transfers are not as well understood [12]. The bulk of relevant literature appeared in the 1990s and early 2000s, suggesting that research has de-prioritised this topic. This presents a missed opportunity to understand the mechanisms involved in policy transfer, especially those between low-income countries and those that are specific to the health sector.

Studies of evidence-based policy-making link to the policy transfer literature by highlighting the types of evidence that currently (or from the perspective of many researchers, should) inform policy, including systematic or scientific research, practical experience and political judgement [13]. Many also recognise that evidence is used in different ways, including instrumentally (using evidence to problem-solve in policy and to improve policy outcomes), conceptually (evidence contributes to knowledge on a particular issue) and symbolically (for example, when evidence is used by politicians to legitimise themselves or to support political claims) [14–19]. Further, it is now widely recognised that policy-makers make decisions in rational and emotional (e.g. political, value-based) ways (using ‘bounded rationality’), which require different forms of evidence [20]. Recommendations for improving the uptake of evidence include pursuing the systematic examination of research that more holistically identifies past lessons and experiences [21]; using research that targets multiple stages of the policy process, for instance, to inform agenda-setting, examining alternatives and outcomes [22]; evaluation of policies that considers political factors [23]; and giving greater attention to the institutional and capacity factors that favour uptake of evidence [24].

Drawing from eight country case studies, this article seeks to supplement existing literature by drawing on the insights and experience of policy-makers in low-income countries and assessing their demand for evidence, how it is met (or not) and what barriers they perceive to exist. It aims to understand how learning has occurred in these case studies of health policy reform and what could be done to strengthen it. It was undertaken by Oxford Policy Management to inform the Bill and Melinda Gates Foundation on priorities for investment in supporting cross-country learning on effective health system policies.

## Methods

The wider study within which the case studies were nested started in early 2017 with three scoping literature reviews focussed on (1) the content of learning across health systems, in terms of which topics comparative health systems literature has addressed since 2000 and using which methods [25]; (2) a review of institutions and platforms that currently exist and aim to facilitate learning across health systems [26]; and (3) international health policy transfer studies [12]. These background reviews and meetings fed into the design and framework for analysis of the case studies, which were overseen by an expert advisory group of researchers. The study was approved by the Ethical Review Committee of the lead UK institution.

The country case studies aimed to answer two research questions, as follows:

1. How do national and sub-national decision-makers access and use ideas and evidence about how to make their health systems work better and where does international evidence fit in that picture?
2. What gaps do national and sub-national decision-makers perceive in their access to appropriate health system evidence in general, and evidence about other countries’ experiences in particular?

Case studies were selected from countries that were categorised as low income in 2000 and performed well in meeting Millennium Development Goal targets by 2015 (had achieved at least 1.5 on the Centre for Global Development’s health score) [27]. From those countries that met these criteria (23 in total), eight were selected as initial case study candidates on the further criteria of geographic spread, inclusion of Anglophone and Francophone African countries, and feasibility of access to appropriate interviewees. The counties selected were Bangladesh, Burkina Faso, Cambodia, Ethiopia, Georgia, Nepal, Rwanda and Solomon Islands. Five of these are currently classified as low income, while three are now lower middle income.

One to two policies or programmes (policy is used as shorthand for both in this article) were chosen per country. The policy selection criteria were simply that the reforms were undertaken within the past decade (to ensure recall by interviewees), and involved significant change to at least one health system block. The studied reforms (described further in Box 1) cover a wide range of reforms, including the Health Extension Programme (HEP) in Ethiopia, the Sector Wide Approach and community clinics in Bangladesh, Health Equity Funds and Special Operating Agencies in Cambodia, hospital privatisation and health sector financing reforms in Georgia, the Integrated Management of Maternal, Neonatal and Childhood Illness programme in Nepal, the Role Delineation Policy in Solomon Islands, health financing reforms, including community-based health insurance (CBHI, the *Mutuelles de Santé*) in Burkina Faso, and CBHI and performance-based financing in Rwanda.

Key informants were selected purposively according to their involvement in the relevant reforms and willingness to be interviewed. The objective of the interviews was to elicit tacit knowledge on the two core research questions – knowledge that is often not documented due to its political and sometimes sensitive nature. A total of 148 key informant interviews were conducted (Table 1). Within these, the largest constituency was technical staff from governmental agencies, followed by technical staff from development partner agencies (bilateral and multilateral organisations that implement and/or fund health interventions).

**Table 1:** Overview of key informants interviewed, by country and constituency

Constituencies	Bangladesh (key informant interview)	Bangladesh (focus group discussion)	Burkina Faso	Cambodia	Ethiopia	Georgia	Nepal	Rwanda	Solomon Islands	Total
Politicians			1		2	2	1	2		8
Technical staff	6		5	3	6	3	5	3	11	42
Development partners	3	5	6	4	2	1	4	4	11	40
Non-governmental organisations	2	7		1	4	2	2	1		19
Academics and consultants	1	10	4	4	3	2	1	9		34
Civil society			3		1			1		5
Total	12	22	19	12	18	10	13	20	22	148

### Data collection and analysis

Data collection was conducted from July to September 2017 and started with the review of published and grey literature on the tracer policies, focusing on the research questions. A semi-structured interview guide was developed and used across the cases, structured according to the policy cycle stages, which had been identified as presenting different issues for evidence use in the international literature. The conceptual framework used to develop the policy stages starts with conceptualisation. This is the beginning of the policy transfer process and refers to the development of the broad idea of the policy itself. Formation and contextualisation refer to the processes by which the key conceptual and operational tenets of policy are concretised and then modified to the social, economic, political, and cultural norms of the country. Internalisation is the process by which a formed policy is accepted and transformed by in-country policy systems. Operationalisation is the process of actually carrying out or implementing the reform. Finally, evaluation refers to critical assessment of any component of the reform [12].

The interview guide included sections and suggested prompts for each of the policy transfer phases, as well as general questions on whether these reform experiences were common to other policies, whether there are particular barriers to learning in policy reform, and whether and how the country had shared its knowledge regarding these reforms with other countries.

Country visits took place over 1-week periods in July to September 2017. Most key informant interviews took place face-to-face, but some were undertaken by phone, as required. In Bangladesh, one focus group discussion was held with ex-government servants, researchers and academics in addition to one-on-one interviews held with key informants. The interviews were conducted by a lead and supporting researcher in each context and each lasted approximately 1 hour. Notes were taken during the interviews and findings were discussed each day between the two researchers. Data was subsequently analysed by both authors individually and then collectively. Data from the document review were primarily used to corroborate and triangulate with information gathered during the interviews, as well as for background information in advance of the interviews.

The framework used for interviewing was also used as a starting point for data extraction and analysis when writing up the case studies, although themes were allowed to emerge inductively as relevant. Once individual case studies were documented, analysis of themes across contexts was produced by the research team, aided by a workshop in October, where commonalities and differences across the case studies were elicited for each topic and discussed by the researchers who had undertaken the country case studies. Findings were presented and discussed at a meeting in Kigali in November 2017, which allowed for further cross-checking of findings. For the drafting of this paper, one researcher analysed across all case studies to present high-level synthetic findings. More detailed evidence is contained in the individual case studies [28–35].

## Results

The findings below are structured according to (1) conceptualisation; (2) uptake or implementation; and (3) further policy development, once a policy has been implemented. We then examine what respondents told us about the mechanisms of learning, which operate at international, regional and national levels. This follows the themes that emerged inductively from the interviews conducted and reflects not just findings on the specific tracer policies but also respondent’s wider comments on learning and evidence use. Finally, we present cross-cutting themes in relation to facilitators and barriers to learning, which are grouped into factors focussed on the demand for and supply of evidence.

### Conceptualisation

All of the reforms either started from or were accompanied by a local recognition of a problem. In relation to the origin of the policies, looking across the eight contexts, five broad models emerged, ranging from least to most home-grown, as follows:

1. In the case of the initial phase of the Integrated Management of Child Illness programme in Nepal, the country was adopting a specific international package, which was more or less standard practice across most countries.
2. In three cases (the Sector-Wide Approach in Bangladesh, health financing reforms in Georgia, and health financing reforms in Burkina Faso), the broad idea behind the policies was initially promoted by major international agencies, but was more actively adopted in the sense of being seen to meet a local need and fit with local contexts.
3. In three cases (Health Equity Funds and contracting in Cambodia, CBHI and performance-based financing in Rwanda, and community clinics in Bangladesh), the policies emerged from a partnership of development partners and government, with ideas being introduced from other contexts but being incubated and developed in substantive ways in-country. Later iterations of Nepal’s Integrated Management of Child Illness followed this path too, through the shift to community-based delivery and the introduction of the package of newborn care.
4. In one case (Role Delineation Policy in Solomon Islands), the idea was developed locally as a means of achieving more equitable, but affordable, health services after a period of ethnic tension. The approach drew on some regional inspiration and technical support from bilateral and multilateral partners.
5. Finally, in the Ethiopian HEP, there was no significant external input, though the policy was influenced in cross-sectoral learning internally from agricultural extension workers in Ethiopia within one state, and later scaled up.

These points illustrate how countries adopted international ideas, but the case studies that were undertaken uncovered many situations in which evidence was not sought, or was altogether ignored. Non-adoption of international ideas and the rejection of advice from other countries had varying consequences. In Ethiopia, the international consensus was antagonistic to community health workers in the late 1990s, when the HEP programme was being developed in Tigray. The government continued to support it, however, as it seemed one of the few feasible ways to reach a dispersed rural population in a context of limited resources and infrastructure. The decision is widely seen to have paid off. Similarly, Cambodia has resisted adopting a clear purchaser-provider split for Special Operating Agencies, despite some international encouragement to do so. Nepal has resisted a number of WHO-recommended adjustments to clinical guidelines, on the basis that they are not in line with wider health system strategy or capacity. Georgia pursued hospital privatisation in the face of cautionary international advice and the legacy of that has been much more mixed.

### Uptake

It is clear from the case studies that the drivers of uptake, or moving ahead with implementation of a policy, are rooted firmly in the local political economy. In the case of Ethiopia, for example, the drivers were historical as well as ideological (the government having recently been engaged in grassroots mobilisation during civil war), combined with political imperatives (the need to deliver basic services to a large, poor population as a new regime) and pragmatism (other options were not feasible with the resources available). Ideological influences, industry lobbying and the powerful role of international agencies (such as the World Bank, during the period of reforms in transitional economies in the late 1990s) are also documented in Georgia, for instance.

Published, peer-reviewed evidence was rarely mentioned as the impetus or main source of information for policy development in the case studies. It was most likely to be consulted for review of clinical protocols, as this is an area in which local contextualisation is regarded as less critical. The influence of published studies is also seen to occur through their dissemination from international agencies such as in the influence of international researchers on healthcare in Burkina Faso and the research on sector-wide approaches that was incorporated by proposals from donor partners in Bangladesh. That said, local evidence being published in an international peer-reviewed journal was said to give it credibility and feed a sense of pride, with both increasing the likelihood of it being acted on.

Robust evidence may be lacking for a policy (like community clinics, in the case of Bangladesh), but if the concept fits well into the socio-political context and enjoys political patronage, then reforms will still be undertaken. The cases of Cambodia and Georgia, where senior politicians made executive policy decisions that were not exactly aligned with the evidence presented, also highlight how governments can set the parameters for when they will or will not over-ride evidence, and how the choice and application of evidence is often ‘purpose-driven’ and predefined by political agendas. In Cambodia, early evidence suggested that contracting services out (to non-government organisations) achieved positive results. The government has been concerned about the sustainability of this option, and adopted a contracting in approach instead. This is an example of some policy options being beyond consideration, even if the evidence may have appeared to be in their favour. This is in contrast to some evidence-informed modifications that have been made by the same government to the operationalisation of Health Equity Funds (though here again, political constraints apply).

### Drivers of policy development (once adopted)

The case studies suggest that internal learning is the key to successful policy development over time. Further, capacities, skills and culture that support good examples in this respect are likely to be linked to the ability to filter experiences from other contexts intelligently.

The case studies illustrated the effective use of annual reviews to assess and improve policy performance (for example, in Rwanda and Ethiopia), adjustment of policies based on local evidence (in Cambodia, Nepal and Rwanda), using national and international routine data sources for monitoring (for example, in Georgia, which used regional comparators for benchmarking), use of evidence from operational research (in Cambodia), and technical assistance to identify the existing – and possible future – cost structures and affordability of interventions (Solomon Islands). Countries like Rwanda, Nepal, Cambodia and Ethiopia were also effective at sharing lessons across sites internally.

By contrast, the role of policy evaluation was much more contested. In some settings, like Bangladesh and Ethiopia, there was resistance to formally evaluating high-priority national programmes, while in others, like Nepal, there were reported tussles over the ownership of the evaluation process. While some countries (e.g. Cambodia) used evaluations actively as a means of lesson-learning and mid-course corrections, many of the apparently successful policies were never formally evaluated, reflecting the higher stakes and more politicised nature of evaluative processes, compared to continuous learning through observation of a policy’s outcomes over time.

### Mechanisms for learning

A wide range of mechanisms that had supported learning processes within and across countries were mentioned by key informants. These are outlined in rough order of frequency, starting with the international ones.

International study tours were the most commonly mentioned mechanism for international learning, used across all eight sites, typically early on in the policy development process and including a variety of constituencies (technical, parliamentary, etc.). These are typically facilitated by development partners and were seen as important, although suggestions for improving their effectiveness (such as better follow-up) were also made.

Country decision-makers and technical staff also use direct relationships with development partner staff to gain advice on topics of interest at all policy stages. Development partners facilitate access to and share ideas and evidence in all settings. Some organisations are widely influential, for example, WHO. Others are seen as offering specific expertise (for example, the World Bank on health financing or International Labour Organization for social protection), though bilateral and multilateral funding agencies are also seen as having their own agendas. Personal relationships with development partner staff are highly important, especially when their presence in-country is long-term, or the country has a small population.

Attending international meetings on specific topics of relevance was also highlighted as influential in five settings (Georgia, Nepal, Solomon Islands, Rwanda and Burkina Faso), particularly regional meetings that focused on a specific, shared problem.

Technical assistance programmes were perceived to be of particular importance in learning about reforms in other countries and in supporting implementation in Bangladesh, Cambodia, Georgia, Solomon Islands, Rwanda and Burkina Faso.

Many countries shared ideas and evidence internally and with external stakeholders such as development partners through routine health system governance structures, such as coordination and technical working groups (highlighted in Cambodia, Georgia, Nepal, Rwanda and Burkina Faso). In some instances, countries systematically established groups to review international published evidence to refine specific health packages (Nepal and Ethiopia).

Capacity-building through formal training or on the job experience also played a role, with countries tending to initially train abroad but gradually develop local capacity and institutions (for example, in Rwanda and Cambodia), also in order to better retain trained staff.

Regional networks also played a role, though these were less frequently mentioned. In the Solomon Islands, regional professional networks may have facilitated idea transfer, including through contractors working across countries, and regional training networks were highlighted as significant. In (former-)francophone African countries (Rwanda and Burkina Faso), influential individual consultants working across countries and community of practice networks were cited as having contributed to the spread of ideas, including through their reports. Burkina Faso was the only context where civil society – in the form of advocacy groups, working with international partners – was cited as having influenced policy uptake.

Within countries, pilot projects supported by international non-governmental organisations played an important role in developing some of the policies (in Rwanda, Cambodia, Nepal and Burkina Faso). Some countries also used domestic study tours and meetings to exchange learning across regions within their country (e.g. Ethiopia).

It was also encouraging that some countries have started to focus on how to share lessons from their own experiences and becoming ‘centres of excellence’ in particular areas, such as Rwanda, which has set up institutions to share lessons on performance-based financing (amongst others), and Ethiopia, which has established an international institute for training and research on rural primary healthcare.

### Facilitators

Facilitators of learning were grouped into those which predominantly affect the demand for evidence, those which are more linked to evidence supply, and finally some which are related to the evidence topic and its presentation.

In relation to demand, having a performance-oriented organisational culture within government was mentioned as a key factor in three settings (Ethiopia, Solomon Islands and Rwanda). Linked to this is proactive identification of evidence needs by the country (highlighted in Bangladesh, Ethiopia, Nepal and Solomon Islands). Growing government financing, confidence and leadership in setting parameters within which evidence is used was highlighted in Cambodia, where a process of growing government leadership was accompanied by a transition in the demand for evidence originating within international organisations to originating within government. This demand can often be focused on internal learning, however, more than seeking evidence from other contexts.

Factors tending to increase confidence in suppliers of evidence included that the latter have in-country staff with embedded knowledge of the health system (highlighted in Bangladesh and Ethiopia). In some cases, authority derives from international agency authority (e.g. for the WHO package), as well as from donor funding and endorsement (Nepal).

In terms of credible evidence supply, this can be facilitated by the development of networks of international and local researchers, producing strong evidence on local policies and building capacity for local analysis (Cambodia). Similarly, consulting groups which maintain deep local roots in the local context while also connecting to international evidence can be effective evidence suppliers (Georgia).

Regional factors were again less prominent but, within West Africa, shared regional identities may play a role, facilitating learning across countries (Burkina Faso), while Nepal has consistently looked to India and Bangladesh for their experiences of community-based care. Shared languages also play a role, for example, francophone African policy, teaching and consulting networks were cited as influential in Rwanda.

The content of the reforms also matters. If reforms are technical and do not imply large structural changes, they will be easier to adopt (Nepal). In terms of the topic and its presentation, evidence is considered by decision-makers when it is politically relevant, accessible and locally applicable (Georgia). It needs to be adapted to the local cultural and geographic context. It is also important that it is presented at the right time in the budget or policy cycle and is communicated in the most acceptable way (for example, oral presentations were highlighted as sometimes preferable in Solomon Islands).

### Barriers

In relation to demand for or use of evidence, cited barriers are grouped into those relating to incentives and those relating to capacity, while on the evidence supply side, capacity and resource factors dominated. Some specific gap areas were also mentioned.

Despite good leadership at the top, lack of accountability for results and weaknesses in supervision at middle management level and below were both barriers to acquiring and implementing learning from others (Solomon Islands). Politicised priorities and institutional constraints to be able to put evidence into effective use were highlighted as barriers in Bangladesh, while fragmentation in the sector and unclear roles was another constraint for operationalisation of policies (Nepal). Civil society was not reported to have played a strong role in the policy cycle in most places (only in Burkina Faso was its influence noted). The lack of an evaluation culture was mentioned in Bangladesh and Solomon Islands, and the issue of decisions being made outside the sector was also raised in the latter. The role of vested interests was highlighted in the Georgia case study, while in others, donor funding was noted to skew priorities. All of these undermine the role and utility of evidence.

Sharing and accessing information can also face barriers. A controlling approach to evidence release was highlighted in Rwanda and Ethiopia and, in some contexts, access to information was even more limited at local (sub-national) levels (e.g. Burkina Faso). Others highlighted the per diem-orientation in relation to participation in meetings, where lesson-learning is further weakened if there is a lack of dialogue and feedback from meetings (Solomon Islands). Sharing of information and evidence is largely personal and unstructured in some settings, rather than being institutional (Burkina Faso). In some places, simple factors like lack of connectivity and ICT skills remain a barrier (Nepal).

Lack of capacity to use evidence well was also mentioned (in Burkina Faso), leading to lack of adaptation of policies from the surrounding region, while in other places (Solomon Islands) participants did not perceive the relevance of evidence from other countries, even evidence from close neighbours (Fiji and Papua New Guinea), which share some similarities but have differences in governance and financing.

On the supply side, a number of countries noted weak in-country capacity to generate evidence (Georgia, Solomon Islands), including the lack of a national institute to perform close-to-policy work; indeed, the Solomon Islands had just one person specifically responsible for research in the Health Ministry, which is not atypical in low-income settings (some have nobody with this role). Having a smaller territory and being geographically isolated may be factors here. Researchers are often unable to be independent because of funding constraints (e.g. Burkina Faso), leading to ad hoc and poor-quality research. Limited national resources to support evidence generation locally were highlighted, especially for health systems research (Ethiopia, Georgia). In some cases, the withdrawal of international support aggravated these challenges (Georgia).

In relation to international agency advice, it is also worth highlighting that pressures and ideas coming from international actors are not always supported by international consensus; indeed, in many cases, international players provide conflicting advice (Georgia), even over technical decisions like on best procedures for Integrated Management of Maternal, Neonatal and Childhood Illness in Nepal. Advice can also be biased by donors’ ‘pet projects’ (Burkina Faso). This is manageable if governments have clear priorities; however, capacity to set clear priorities is itself commonly a barrier in these settings.

Some noted that, while there is relatively good access to policy documents and general guidance online, it is harder to find operational information on how to implement specific reforms (Ethiopia, Rwanda). Furthermore, it was noted by several respondents that the substantial amount of online information may be useful, yet it is difficult to access and time-consuming to sift through. There is therefore a demand for a brokerage function that would identify high quality, implementable information from other studies and reports. Some also felt that there was a lack of access to practical information, such as regional drug prices or trends in non-communicable diseases (Solomon Islands), while language barriers and limited access to journals remain challenges in some areas such as in Burkina Faso.

## Discussion

Many of the case study findings are consistent with the broader literature on health policy transfer in low- and middle-income countries [36, 37]. Both case studies and the literature illustrate that evidence is used in conceptualisation through the identification of a problem or policy need, facilitated by relationships that exist through policy networks, and sometimes through advocacy of international agencies, and is facilitated by the alignment of goals between relevant stakeholders [9–11, 38]. However, the case studies illuminate many aspects of health policy transfer that are either differently or under-represented in current literature. These aspects include political economic factors, how policies are implemented and the types of evidence that are used to inform implementation, and the kinds of practical mechanisms that are useful for policy-makers. The mechanisms highlighted are very varied but those which are most frequently cited – study tours and face-to-face interaction with development partners – highlight the importance of experiential learning, which allows for sharing of not just technical but also political insights. This article also complements existing literature by starting from a national and sub-national perspective (not the ‘push’ approach adopted by much of the literature on how international actors can promote evidence uptake, which tends to take a normative stance) and using a range of low- and middle-income settings to draw a broader analysis.

By starting from actual policy decisions (rather than from questions about use of international evidence), we find that many of our studied policies were home-grown or at least heavily home-incubated (for example, in Ethiopia, Rwanda, Bangladesh, Nepal and Cambodia). We can speculate that this may link with their subsequent good performance, either due to higher ownership and/or a correlation between the capacity to innovate and the capacity to manage implementation well. Social factors determining the effectiveness of policies, such as cultural norms changing how maternal health policies in Nepal and community clinics in Bangladesh are implemented, were understood by policy-makers. As a result, formative and technical recommendations from international agencies that conflict with these norms are generally rejected or adjusted by policy-makers.

The commitment of ‘national elites’ to policy transfer is commonly cited throughout both literature and the case studies as crucial for the success of policy implementation [39, 40]. Elites may consist of politicians, leaders of government agencies and organisations, as well as individuals who are employed by or participate in their home government but interact with international policy communities [40]. Discussions between international actors and national elites around the Sector-Wide Approach in Bangladesh and the formation of strategic plans in the health sector in Cambodia, as well as the integral role of policy-makers in small countries like the Solomon Islands, with a high turnover of development partner staff and limited numbers of senior level officials, demonstrate that buy-in from in-country policy-makers is crucial for reforms to be adopted and subsequently for resources to be mobilised around scaling up those reforms (see also Shroff et al. [41]).

International agencies are often cited as important since they mobilise interest and resources around issues that affect when and how a policy is conceptualised [42]. Bennett et al. [11] describe the role of agencies as being between advocates and neutral facilitators in the transfer of policy. This is a theme which emerges throughout the literature as agencies either impose or neutrally act as the medium through which policy is transferred. While most criticism of international agencies in the literature centres around the issue of coercion and how agencies and their financing have been used as a means for wealthy countries to shape policy formation for their own agendas [38, 43], the case studies present a more nuanced understanding of the role of international agencies as being influencers rather than controllers of policy conceptualisation, uptake and development. The case studies acknowledge that international agencies have their own mandates and agendas that, in some cases, differ from the governments they work with; however, the impact of agencies is met with the motivations of key in-country decision-makers. This greater agency given to domestic actors may reflect the make-up of our respondents to some extent, although more likely it relates to the country selection and the bias towards ‘strong performers’. Country leadership is also not stable over time – cases like Cambodia have seen a growth from a low base to current greater national confidence. These stages will very much influence demand for and use of international and local evidence.

The case studies overwhelmingly suggest that local political and economic factors determine when and what international evidence is used and whether that use is instrumental or conceptual (symbolic use was not raised in these case studies) [14–16]. Political power often supersedes the influence of international agencies and national technical elites, having earned support from the wider public and established social groups [44]. Unlike other policy transfer stakeholders, political parties have the ability to manoeuvre both public and private (e.g. corporate) interests [45]. In addition, other stakeholders often rely on political support to influence policy decisions, including those who provide financial, programmatic, and technical services [46–48].

It is also striking that conflict or crisis had propelled reforms in the majority of our case studies (Rwanda, Ethiopia, Solomon Islands, Nepal, Bangladesh and Cambodia), presumably creating the need and motivation to innovate, as well as a momentum to reduce inertia, challenge path dependencies and willingness to risk policy errors [35]. Resource constraints were also seen as having encouraged creativity in adopting new policies in some cases.

The existence of policy networks is another mechanism that is widely identified as important in conceptualisation by both published studies and the case studies. Policy networks consisting of formal or informal relationships between governments and other policy stakeholders [49] are understood to be useful for promoting dialogue and learning between stakeholders [11, 50, 51], and are enhanced by political and social connections between decision-makers and other actors [38, 39]. As would be expected, the case studies demonstrate that, while the influence of external information is typically stronger at earlier stages of the policy cycle, i.e. conceptualisation, implementation is strongly influenced by internal learning within policy networks, although external actors, especially consultants and technical assistants, remain important for the operational stage. The case studies point towards consistent dialogue between stakeholders as a mechanism of evidence uptake in conceptualisation, for instance, through discussions and consultations which led to health financing reforms in Georgia, the facilitation of learning through professional connections between officials and development partners in the Solomon Islands, and interactions between health officials in regional meetings and study tours in Burkina Faso and Rwanda.

Other studies on health policy transfer suggest that evaluation is needed to improve dissemination of progress in health policy reform and implementation [52], follow-up and management [53], alignment of policy goals and messages across stakeholders [54], and quality of health services provided through transfer [55]. The case studies in Cambodia, Nepal, Burkina Faso, and Rwanda show that evaluation can inform conceptualisation of policy by identifying weaknesses in health policies and informing policy development from pilot project outcomes and impact evaluations. However, in some cases, evaluations were blocked for political reasons. As evaluations present a more summative judgment, they are potentially more threatening to high profile policies than feedback from continuous monitoring.

Our findings highlight the importance of continuous learning and many positive examples of institutions which are doing this in different contexts. This is an important supplement to current literature, which does not provide much insight into how continuous learning affects uptake of evidence in health policy transfer. Most of the findings highlight the importance of developing the domestic incentives and demand for evidence – areas of gap in supply of evidence were reportedly more minor by comparison, as seen from the national level, though this is not to deny on-going access barriers. Smith et al. [56], for example, analyse more than 3000 papers in almost 1000 journals dealing with global health, and conclude that only 39% of papers published in a journal have open access, and 42% of scholarly articles require a subscription, although there is an increasing wealth of evidence available in grey and open-access sources.

The case studies illustrate how evidence that is used to inform policy is not ‘systematic’ in nature, in that evidence is often not systematically collected, examined or applied. This supports the views of most practitioners and many academics [23] that the ideal type of evidence-based policy – in which policy-makers are comprehensively rational, have the ability to systematically rank policy alternatives, and prioritise robust and critically appraised evidence – is unrealistic. Cairney and Oliver [20] suggest that researchers can be most effective when combining the principles of evidence and governance. They argue that the weight of value-driven arguments can be just as important, if not more so, to policy-makers than the importance of evidence and, therefore, evidence could be packaged to accommodate policy-makers’ social, political and ideological predispositions and motives [20]. The case studies illustrate the variety of forms and processes through which evidence is used, and imply that evidence is best conceptualised as one element feeding into decisions, which are dominated by the interests and outlooks of the most influential actors. Perceived fit to local needs and context is key.

There are some important limitations to note, which include that the countries were selected as relatively strong performers which had undertaken some form of significant health system reform in recent years. The selection allowed for the study of how evidence had, or not, informed policies. However, the findings may not be representative of a wider set of countries that may be less active in policy innovation. It should also be noted that each case study was conducted in a limited time, and thus not all perspectives are reflected and included. We can therefore regard the findings as a rich snapshot, rather than as a complete account. We also highlight our inductive approach to analysis, which meant that a structured comparison of learning across systems within a pre-determined theoretical framework was not undertaken.

However, the article can inform the future development of a conceptual framework for learning health systems, which should include not just internal factors (such as alignment of actors, incentives, capacities and resources) but also openness to and mechanisms for filtering international experiences and evidence (personal, organisational and institutional, explicit and tacit, strategic) by different actors and for different purposes (strategic, political and technical). Existing frameworks do not adequately reflect the agency we found for local decision-makers, as much of the focus is on ‘push’ models, such as policy transfer (which emphasises the transfer of specific ideas) and evidence-based policy-making (which emphasises getting research into practice), both neglecting a more active role of demanding, shaping and co-creating knowledge in the local arena.

## Conclusion

This article reviews the experiences of eight low- and low–middle-income countries which have adopted health system reforms in the past two decades. Using key informant interviews with those directly engaged in the reforms at different periods of time, it probes whether and how international policy transfer occurred, how evidence informed the different stages of the policy cycle, what mechanisms were effective for learning and what barriers and facilitators were perceived by the participants. Extra focus was placed on unpacking the role of learning from other countries throughout the reform process. The findings emphasise the agency of national and sub-national players and the importance of developing local institutions for gathering, filtering and sharing evidence, locally as well as south–south. Developing demand for and capacity to use evidence appears more significant (in terms of current barriers) than augmenting the supply internationally, although specific gap areas were identified by respondents, especially in relation to more operational and practical questions. The case studies also highlighted that, beyond an initial sharing of information, a lot of work is needed to adequately contextualise and internalise ideas in a new setting. The overall learning process (including conceptualisation, uptake and development) is a long-term and complex endeavour, in some cases taking 15 to 20 years before a lesson can be said to be ‘in action’ at a national level. The findings also highlight the importance of the local political economy in setting the parameters within which evidence is considered and the importance of trusting relationships between national and international individuals and organisations. Finally, we highlight the need for a theoretical framework within which to further analyse learning across health systems.

Box 1: Background on case study countries and policies **Bangladesh**

Recent health sector reforms in Bangladesh commenced with the Health and Population Sector Strategy developed by government and donor partners in 1997, resulting in the pooling of donor funds through a Sector-Wide Approach. The introduction of one-stop services through Community Health Clinics to replace domiciliary services provided by Family Planning Services field staff were also established in 1998 to herald a major shift in family planning services, from door-to-door to clinic based [34].

**Burkina Faso**

Community-based health insurance (*Mutuelles de Santé*), as a health financing policy intervention, has had a long history in Burkina Faso, from the first experiments in the late 1980s to the 288 schemes identified in 2013. Moreover, the community-based health insurance ‘movement’ is said to have given rise to significant policy initiatives such as the planned universal health insurance (*Assurance Maladie Universelle*) [33].

**Cambodia**

Cambodia’s health sector has been innovative. Among many initiatives that have accompanied the longer-term process of health reform that began in the mid-1990s, two in particular have attracted significant international attention. The Health Equity Fund (which was initiated in 2000) is now a nationwide social health protection scheme, delivering publicly provided health services to the poorest one-fifth of the population. On the supply side, the development of a unique form of contracting in the delivery of public health services (launched in different forms in the mid-1990s) has begun to produce observable results in the management of health service delivery [32].

**Ethiopia**

One of the policies credited with making a substantial contribution to progress towards achieving the health-related Millennium Development Goals 4, 5 and 6 in Ethiopia is the government’s flagship Health Extension Program. Launched in 2003 and gradually scaled up nationwide, the Health Extension Program helped develop a new cadre of paid female community health workers, supported by volunteers at community level and contributed to universal access to primary health services in rural areas [33].

**Georgia**

Georgia has introduced extensive health sector reforms and made significant progress against the Millennium Development Goals by 2010. However, while some of the reforms were driven by international best practice, closely resembling developments in the region (e.g. health financing reforms in 1997–2003 aiming at introduction of Social Health Insurance, and later reforms from 2012 targeting Universal Health Coverage), others were home-grown and sometimes quite radical (e.g. hospital reforms in 2006–2012, resulting in privatisation of over 70% of public hospitals in a poorly governed environment, with subsequent implications for costs and quality of services) [31].

**Nepal**

Despite the constraints, Nepal made substantial progress in reaching the Millennium Development Goals, especially in reducing child mortality. Community- and facility-based health interventions focused on child health such as Integrated Management of Childhood Illness (now known as Integrated Management of Maternal, Neonatal and Childhood Illness), vitamin A supplementation, immunisation, and deworming programmes contributed to achieving the reduction. This was facilitated by a network of 50,000 female community health volunteers that played an important role in promoting health and reducing the gap between the community and the health facility [29].

**Rwanda**

Rwanda achieved substantial population health improvements and is particularly known for what is widely considered to be a successful introduction of community-based health insurance and performance-based financing, alongside wider health reforms including more effective aid coordination [30]. Introduced from the mid-1990s to early 2000s, Rwanda implemented community-based health insurance and performance-based financing – targeting demand- and supply-side barriers respectively – significantly more effectively and at a larger scale than any other low-income country [30].

**Solomon Islands**

The Role Delineation Policy in the Solomon Islands was developed to better define the range and level of services – or packages of care – to be delivered to populations across the country. It is designed to be a strategic and system-wide reform, delivering needed services, particularly to rural areas, in a way that is financially and institutionally sustainable. Over the 15 years through which it has been developed, the Role Delineation Policy has become a central part of policy for improved health services [28].

## Abbreviations

CBHI: Community-Based Health Insurance
HEP: Health Extension Programme

## References

[1] United Nations Population Fund (2014). State of the World Population 2014: The Power of 1.8 Billion, Adolescents, Youth and the Transformation of the Future.

[2] Gottret P, Schieber G (2006). Health Financing Revisited: A Practitioner’s Guide.

[3] UN General Assembly (2015). Resolution A/RES/70/1. Transforming Our World: The 2030 Agenda for Sustainable Development. Seventieth United Nations General Assembly, New York, 25 September 2015.

[4] Dieleman J, Murray CJL, Haakenstad A, Graves C, Johnson E, Templin T, Birger M, Singh L, Leach-Kemon K (2015). Financing Global Health 2014: Shifts in Financing as the MDG Era Closes.

[5] Dieleman J, Murray CJL, Haakenstad A, Leach-Kemon K, Baral R, Graves C, Johnson E, Templin T (2014). Financing Global Health 2013: Transition in an Age of Austerity.

[6] Alshamsan R, Lee JT, Rana S, Areabi H, Millett C (2017). Comparative health system performance in six middle-income countries: cross-sectional analysis using World Health Organization study of global ageing and health. J R Soc Med.

[7] OECD (2010). Health Care Systems: Getting More Value for Money.

[8] Peters DH, Garg A, Bloom G, Walker DG, Brieger WR, Rahman MH (2008). Poverty and access to health care in developing countries. Ann N Y Acad Sci.

[9] Dolowitz D, Marsh D (1996). Who learns what from whom: a review of the policy transfer literature. Polit Stud.

[10] Evans M, Davies J (1999). Understanding policy transfer: a multi-level, multi-disciplinary perspective. Public Adm.

[11] Bennett S, Dalglish SL, Juma PA, Rodriguez DC (2015). Altogether now… understanding the role of international organizations in iCCM policy. Health Policy Plan.

[12] Jensen C, McPake B, Jones A (2017). Learning for Across Health Systems: Literature Review of International Health Policy Transfer Processes.

[13] Head BW (2008). Three lenses of evidence-based policy. Aust J Public Adm.

[14] Sanderson I (2002). Making sense of ‘what works’: evidence based policy making as instrumental rationality?. Public Policy Adm.

[15] Amara N, Ouimet M, Landry R (2004). New evidence on instrumental, conceptual, and symbolic utilization of university research in government agencies. Sci Commun.

[16] Boswell C (2009). The Political Uses of Expert Knowledge: Immigration Policy and Social Research.

[17] Pelz D, Yinger MJ, Cutler S (1978). Some expanded perspectives on use of social science in public policy. Major Social Issues: A Multidisciplinary View.

[18] Weiss CH (1979). The many meanings of research utilization. Public Adm Rev.

[19] Lomas J, Brown AD (2009). Research and advice giving: a functional view of evidence-informed policy advice in a Canadian Ministry of Health. Milbank Q.

[20] Cairney P, Oliver K (2017). Evidence-based policymaking is not like evidence-based medicine, so how far should you go to bridge the divide between evidence and policy?. Health Res Policy Syst.

[21] Pawson R (2002). Evidence-based policy: the promise of ‘realist synthesis. Evaluation.

[22] Solesbury W (2001). Evidence Based Policy: Whence it Came and Where it’s Going. Working Paper.

[23] Sanderson I (2002). Evaluation, policy learning and evidence-based policy making. Public Adm.

[24] Witter S, Kardan A, Scott M, Moore L, Shaxton L (2017). Generating demand for and use of evaluation evidence in government health ministries: lessons from a pilot programme in Uganda and Zambia. Health Res Policy Syst.

[25] Binachon B, Awosusi A, Hanson K, Ensor T, Jones A (2017). Landscaping Review Part 1: Review of Comparative Health Systems Literature.

[26] Witter S, Anderson I, Bhandari N, Jones A (2017). Landscaping Review Part 2: What Types of Institutions Currently Facilitate Learning between Countries about Improving Health Systems?.

[27] Leo B, Barmeier J (2010). Who are the MDG Trailblazers? A New MDG Progress Index.

[28] Anderson I, Gilbert K (2017). Learning for Action Across Health Systems, Case Study of Solomon Islands.

[29] Bhandari NN, Jones A (2017). The Role of Learning from other Countries in Nepali Health System Reform, Focusing on CB-IMCI/NCP/IMNCI Reform: Learning for Action Across Health Systems, Case Study.

[30] Brikci N, Binachon B (2017). The Role of Learning from other Countries in CBHI and PBF Reforms in Rwanda: Learning for Action Across Health Systems, Case Study.

[31] Chanturidze T, Jensen C (2017). The Role of Learning from Other Countries, Case Study, Georgia: Learning for Action Across Health Systems, Case Study.

[32] Jones A, Annear P (2017). The Role of Learning from Other Countries in Cambodian Health System Reform, Focusing on Health Equity Funds and Innovative Contracting: Learning for Action Across Health Systems, Case Study.

[33] Lievens T, Binachon B (2017). The Role of Learning from Other Countries in Health System Reforms in Burkina Faso, Focusing on Healthcare Financing Reforms: Learning for Action Across Health Systems, Case Study.

[34] Raichowdhury S, Jensen C (2017). The Role of Learning from Other Countries in Bangladesh: Learning for Action Across Health Systems, Case Study.

[35] Witter S, Awosusi A (2017). Ethiopia and the Health Extension Programme: Learning for Action Across Health Systems, Case Study.

[36] Lavis JN, Oxman AD, Moynihan R, Paulsen EJ (2008). Evidence-informed health policy 1: synthesis of findings from a multi-method study of organizations that support the use of research evidence. Implement Sci.

[37] Koon AD, Rao KD, Tran NT, Ghaffar A (2013). Embedding health policy and systems research into decision-making processes in low- and middle-income countries. Health Res Policy Syst.

[38] Clark BY (2009). Policy adoption in dynamic international environments: evidence from national aids programs. Public Adm Dev.

[39] Lee K, Walt G (1995). Linking national and global population agendas: case studies from eight developing countries. Third World Q.

[40] Robinson RS (2012). Negotiating development prescriptions: the case of population policy in Nigeria. Popul Res Policy Rev.

[41] Shroff ZC, Tran N, Meessen B, Bigdeli M, Ghaffar A (2017). Taking results-based financing from scheme to system. Health Syst Reform.

[42] Ogden J, Walt G, Lush L (2003). The politics of ‘branding’ in policy transfer: the case of DOTS for tuberculosis control. Soc Sci Med.

[43] Banerji D (1999). A Fundamental Shift in the Approach to International Health by WHO, UNICEF, and the World Bank: Instances of the Practice of “Intellectual Fascism” and Totalitarianism in some Asian Countries. Int J Health Serv.

[44] Hunter W, Brown DS (2000). World Bank directives, domestic interests, and the politics of human capital investment in Latin America. Comp Pol Stud.

[45] Gomez EJ, Ruger JP (2015). The global and domestic politics of health policy in emerging nations. J Health Polit Policy Law.

[46] Minoletti A, Galea S, Susser E (2012). Community mental health services in Latin America for people with severe mental disorders. Public Health Rev.

[47] Perez-Ferrer C, Lock K, Rivera JA (2010). Learning from international policies on trans fatty acids to reduce cardiovascular disease in low- and middle-income countries, using Mexico as a case study. Health Policy Plan.

[48] Wonodi CB, Privor-Dumm L, Aina M, Pate AM, Reis R, Gadhoke P, Levine OS (2012). Using social network analysis to examine the decision-making process on new vaccine introduction in Nigeria. Health Policy Plan.

[49] Rhodes RAW, Moran M, Rein M, Goodin RE (2008). Policy network analysis. The Oxford Handbook of Public Policy.

[50] Buchan J, Couper ID, Tancharoensathien V, Thepannya K, Jaskiewicz W, Perfilieva G, Dolea C (2013). Early implementation of WHO recommendations for the retention of health workers in remote and rural areas. Bull World Health Organ.

[51] Pallas SW, Nonvignon J, Aikins M, Ruger JP (2015). Responses to donor proliferation in Ghana’s health sector: a qualitative case study. Bull World Health Organ.

[52] Brundage SC, Carty L, Elias CJ, Fleischman J, Morrison JS (2011). On the Ground with the Global Health Initiative: Examining Progress and Challenges in Kenya.

[53] Jashi M, Viswanathan R, Ekpini R, Chandan U, Idele P, Luo C, Legins K, Chatterjee A (2013). Informing policy and programme decisions for scaling up the PMTCT and pediatric HIV response through joint technical missions. Health Policy Plan.

[54] Ngoasong MZ (2009). The emergence of global health partnerships as facilitators of access to medication in Africa: a narrative policy analysis. Soc Sci Med.

[55] Sundby J (2014). A rollercoaster of policy shifts: global trends and reproductive health policy in The Gambia. Glob Public Health.

[56] Smith E, Haustein S, Mongeon P, Shu F, Ridde V, Lariviere V (2017). Knowledge sharing in global health research: the impact, uptake and cost of open access to scholarly literature. Health Res Policy Syst.

